# Frailty in Rheumatic Diseases

**DOI:** 10.3389/fimmu.2020.576134

**Published:** 2020-10-29

**Authors:** Francesca Motta, Antonio Sica, Carlo Selmi

**Affiliations:** ^1^ Division of Rheumatology and Clinical Immunology, Humanitas Clinical and Research Center– IRCCS, Rozzano, Italy; ^2^ Department of Biomedical Sciences, Humanitas University, Rozzano, Italy; ^3^ Humanitas Clinical and Research Center - IRCCS - Laboratory of Molecular Immunology, Milan, Italy; ^4^ Department of Pharmaceutical Sciences, University of Piemonte Orientale “A. Avogadro”, Novara, Italy

**Keywords:** rheumatic diseases, osteoarthritis, rheumatoid arthritis, connective tissue diseases, vasculitis, frailty, frailty index, inflammaging

## Abstract

Frailty is a syndrome characterized by the decline in the physiologic reserve and function of several systems, leading to increased vulnerability and adverse health outcomes. While common in the elderly, recent studies have underlined the higher prevalence of frailty in chronic diseases, independent of age. The pathophysiological mechanisms that contribute to frailty have not been completely understood, although significant progresses have recently been made. In this context, chronic inflammation is likely to play a pivotal role, both directly and indirectly through other systems, such as the musculoskeletal, endocrine, and neurological systems. Rheumatic diseases are characterized by chronic inflammation and accumulation of deficits during time. Therefore, studies have recently started to explore the link between frailty and rheumatic diseases, and in this review, we report what has been described so far. Frailty is dynamic and potentially reversible with 8.3%–17.9% of older adults spontaneously improving their frailty status over time. Muscle strength is likely the most significant influencing factor which could be improved with training thus pointing at the need to maintain physical activity. Not surprisingly, frailty is more prevalent in patients affected by rheumatic diseases than in healthy controls, regardless of age and is associated with high disease activity to affect the clinical outcomes, largely due to chronic inflammation. More importantly, the treatment of the underlying condition may prevent frailty. Scales to assess frailty in patients affected by rheumatic diseases have been proposed, but larger casuistries are needed to validate disease-specific indexes, which could allow more accurate prognostic estimates than demographic and disease-related variables alone. Frail patients can be more vulnerable and more difficult to treat, due to the risk of side effects, therefore frailty should be taken into account in clinical decisions. Clinical trials addressing frailty could identify patients who are less likely to tolerate potentially toxic medications and might benefit from more conservative regimens. In conclusion, the implementation of the concept of frailty in rheumatology will allow a better understanding of the patient global health, a finest risk stratification and a more individualized management strategy.

## Introduction

Life expectancy has markedly increased worldwide during the last decades, mainly due to medicine progresses (1). Therefore, the number of older adults has increased and the population of people aged over 60 years is estimated to double within the next 30 years (2). The risk to develop a chronic disease with cognitive and physical impairment increases with aging and is expected to represent the next major medicine challenge. Nonetheless, individuals may have the same chronological but very different biological ages (3), depending on genetic, biological, and environmental factors as well as physical, psychological, and social determinants. In the attempt to define the more vulnerable subjects, the concept of frailty was introduced in the 1950s and 1960s first in geriatric medicine, but only in the recent years it gained attention by the scientific community (2). At the earlier stages of research, the term frailty was used as a synonymous of aging, disability, or comorbidity, since these states are often overlapping. However, the concepts are different: advanced age does not necessarily mean vulnerability, disability means loss of function and comorbidity implies two or more diseases (4). As a general rule, frailty indicates a decreased physiological reserve and compromised capacity to maintain homeostasis as a consequence of time-related, multiple, accumulated deficits.

A physiological decline in organ function occurs invariably with aging but this process is aberrant in frailty to involve multiple systems, leading to the dysregulation of the homeostatic balance with vulnerability in response to minor stressor events. This brings to further reserve decrease, in a vicious cycle (5, 6). Possible etiologic factors include genetic and epigenetic mechanisms (7, 8), metabolic and environmental stressors and acute or chronic diseases (9). As a consequence, multiple processes are altered, with the major role of the immune, musculoskeletal, endocrine, and neurological systems. Frailty is due to the impaired function of these inter-related physiological systems, lowering their reserve under the threshold needed to compensate changes (6).

Despite a standard definition of frailty remains lacking, the most inclusive is the one provided by the WHO: i.e. a clinically recognizable state in which the ability to cope with everyday or acute stressors is compromised by an increased vulnerability brought by age-associated decline in physiological reserve and function across multiple organ systems (2).

Frail adults are at increased risk of death and negative health outcomes, including falls, fractures, disability, cognitive decline and poor quality of life, with an impact on medical costs and health care resources (10). The prevalence of frailty in the general population aged 65 years and older can range between 4% and 59%, depending on the criteria used and about 25% of people aged 80 years or older are considered to be frail (11). Of note, a high prevalence of frailty has also been found in selected populations with specific diseases or chronic conditions, not necessarily associated with age, such as cirrhosis (12), HIV infection (13), end-stage renal disease (14) and heart failure (15). Nonetheless, frailty is not invariably age-related, as chronic conditions can cause vulnerability and increase the risk of negative outcomes. Rheumatic diseases affect subjects at any age and the chronic condition might reduce the physiological reserve and increase vulnerability. In this field, studies on frailty are limited and the implementation of this concept could provide further information on the overall health status of patients.

## Frailty Measurement

Several frailty measurements have been created for clinical and research purposes (16, 17). **Table 1** illustrates the most widely used tools (26), with Fried’s frailty phenotype and the frailty index of accumulated deficits being the most commonly employed. The Fried’s frailty phenotype is also known as the Cardiovascular Health Study (CHS) Index, from the study that originally used it (18) and includes physical features, or “phenotype,” defining frailty as the presence of three or more of the following: (i) weight loss: body mass index (BMI) ≤18.5 or self-reported unintentional weight loss ≥4.5 kg in the previous year; (ii) exhaustion: self-reported; (iii) slowness: walking 4 m in 6.13 sec or more for height ≤159 cm or in 5.25 s or more for height >159 cm; (iv) weakness: grip strength (measured with a dynamometer) ≤17 kg for BMI ≤23 kg/m^2^, ≤17.3 kg for BMI 23.1 to 26 kg/m^2^, ≤18 kg for BMI 26.1-29 kg/m^2^, or ≤21 kg for BMI >29 kg/m^2^; (v) low physical activity: measured with the International Physical Activity Questionnaire (27). Subjects are considered as “pre-frail” when only one or two criteria are met, whereas subjects are considered as not frail or robust in the absence of any of the aforementioned factors. The Fried index is predictive of adverse clinical outcomes, including mortality (28, 29). However, its widespread clinical application is limited by the inclusion of measurements not routinely used in clinical practice and the exclusion of psychosocial components or cognitive impairment. Nonetheless, the Fried’s frailty phenotype has been widely applied and validated (30–32).

**Table 1:** Comparison of different frailty scales.

Index	Country of origin	Items	Frailty definition	Time (min) to assess frailty	Special equipment needed for measurement	Ref
**Fried**’**s Frailty Phenotype**	USA	5	Frailty ≥3; pre-frail 1–2; Robust=0	<10	yes	(18)
**FI-CD**	Canada	30+	Continuous score; frailty cut-off >0.25	≈30	no	(19)
**Gill Frailty Measure**	USA	2	Moderately frail if rapid gait>10 s or could not stand from the chair. Severely frail if meet both criteria.	<10	no	(20)
**Frailty/Vigor Assessment**	USA	13	Score: 0–9 frail attributes. 0–4 vigorous attributes Frail: ≤1 vigorous and ≥4 frail attributes. Vigorous: ≥3 vigorous and ≤2 frail attributes. Transitional: neither frail nor vigorous.	<20	no	(21)
**CSHA-CFS**	Canada	7	Moderately frail: 6 Severely frail: 7	<20	no	(22)
**Brief Frailty Instrument**	Canada	5	Index score range 0–5 (high score=high risk): 4 categories: 0; 1; 2; ≥3	<20	no	(23)
**Vulnerable Elders Survey**	Japan	13	Frail if score ≥3	<15	no	(24)
**FRAIL**	USA	5	Frailty ≥3; pre-frail 1–2; Robust=0	<10	no	(25)

FI-CD, Frailty Index of Accumulated Deficits; CSHA-CFS, Canadian Study of Health and Aging Clinical Frailty Scale; FRAIL, Fatigue, Resistance, Ambulation, Illness and Loss of Weight Index.

As problems accumulate over time, less or more rapidly, frailty might be seen as the sum of deficits, leading to increased vulnerability. The Frailty Index (FI) of Cumulative Deficits (FI-CD) was developed by Rockwood, Molginer and Mitnitski (19) as part of a 5-year prospective cohort study (n=10263) conducted in elderly people in Canada (mean age 82 years). Ninety-two baseline variables of symptoms (eg, low mood), signs (based on physical exam), abnormal laboratory values, disease states and deficits were considered and the presence or absence of each variable is set as proportion of the total (eg, 15 deficits present out of 92 variables gives a frailty index of 15/92=0.16). It has been shown that the list of variables can be reduced to 30, without missing predictive validity (33). The exact list for inclusion in the FI-CD does not specifically matter. In fact, a process for creating a cumulative deficits frailty index has been defined (34) and variables can be selected if they meet the following criteria: (i) associated with health status, (ii) prevalence increasing with age; (iii) not saturated too early, (iv) reflective of a range of physiological systems, (v) if it is to be used serially on the same people, the items need to be the same. On the contrary, when considering frailty of different groups of people, different indexes can be compared, as although items may differ, results are similar.

The FI-CD has a high predictive value for adverse clinical outcomes (35) and it seems that its total score, rather than type of health deficit, may better predict adverse outcomes (36). An upper limit is believed to be set at 0.67, beyond which the likelihood of survival is minimal (37). The FI-CD has also been applied to the Survey of Health, Ageing and Retirement (SHARE) study in Europe, where it is termed the SHARE-FI (38). This continuous FI allows greater ability to discriminate between different degrees of frailty, compared to the Fried phenotype (39). The main limitation is the time-consuming process to calculate the index. Nonetheless, this two frailty scales show significant overlapping in their identification of frailty and in the ability to predict disability and mortality (40).

## Inflammation and Frailty

The etiology of frailty remains poorly understood but it is likely that numerous factors are involved in the pathogenesis and more systems are affected, leading to the loss of homeostasis and to a vicious cycle with further decrease of the physiological reserve. The genetic background that can influence frailty has been studied, and genes found to be associated with frailty are related to inflammation, muscle function, glucose and lipid metabolism, hypothalamic-pituitary-adrenal axis function, apoptosis, and homeostasis, such as genes encoding for methionine synthase, fibronectin or transcriptional factors (41). Although genetics seems to have an impact on frailty, acquired and environmental factors probably have the highest contribution, also through epigenetic modifications (42). On these bases, a multi-systemic dysregulation occurs and multiple biologic mechanisms are altered.

The immune system has a key role in this context and patients affected by rheumatic diseases might thus have an additional risk factor for frailty. It has been shown that chronic inflammation contributes to the development of frailty, both directly and indirectly through other processes. A direct association between frailty and increased total white blood cell count has been found (43, 44). Moreover, frailty is associated with a pro-inflammatory T-lymphocyte phenotype, as an increased count of cluster of differentiation (CD)8+/CD28− T cells and C-C chemokine receptor type 5 (CCR5)+ T cells has been demonstrated (45, 46). The analysis of monocytes gene expression has further shown the upregulation of several stress-responsive inflammatory pathway genes in frail individuals (47), while several pro-inflammatory molecules are associated with frailty. Elevated serum concentration of interleukin (IL)-6 was observed in frail elderlies (43, 48, 49), and higher IL-6 levels were produced by peripheral blood cells (PBMCs) from frail subjects when stimulated by lipopolysaccharide (LPS), compared with non-frail adults LPS-stimulated PBMCs (50). In IL-10 deficient mice with features mimicking human frailty, such as muscle weakness, IL-6 was significantly higher compared to age- and gender-matched C57BL/6J control mice (51). C-reactive protein (CRP), tumor necrosis factor-α and CXC chemokine ligand-10, a potent pro-inflammatory mediator, are also elevated in frail older adults (47, 49, 52). Elevated levels of neopterin, a marker for immune activation mediated by monocytes and macrophages, are associated with frailty in community-dwelling older adults independently of IL-6 levels, suggesting that immune activation plays a key role in the pathogenesis of frailty. The underlying factor that stimulates the immune system activation can be an autoimmune process, as happens in rheumatic diseases, or a malignancy, or a persistent infection (53). In fact, positive anti-CMV immunoglobulin G titers have been found to be associated with frailty (54, 55).

The impaired immune system associated with frailty might function adequately in the quiescent state but fails to respond appropriately to a stressor event. Evidence suggests that in frailty an abnormal, low-grade inflammatory response persists for a long period after removal of the initial inflammatory stimulus and is hyper-responsive to further stimuli. In fact, acute episodes of illness or exacerbation of chronic conditions may accelerate the development of frailty or worsen its clinical presentation and adverse outcomes (5).

**Figure 1:**
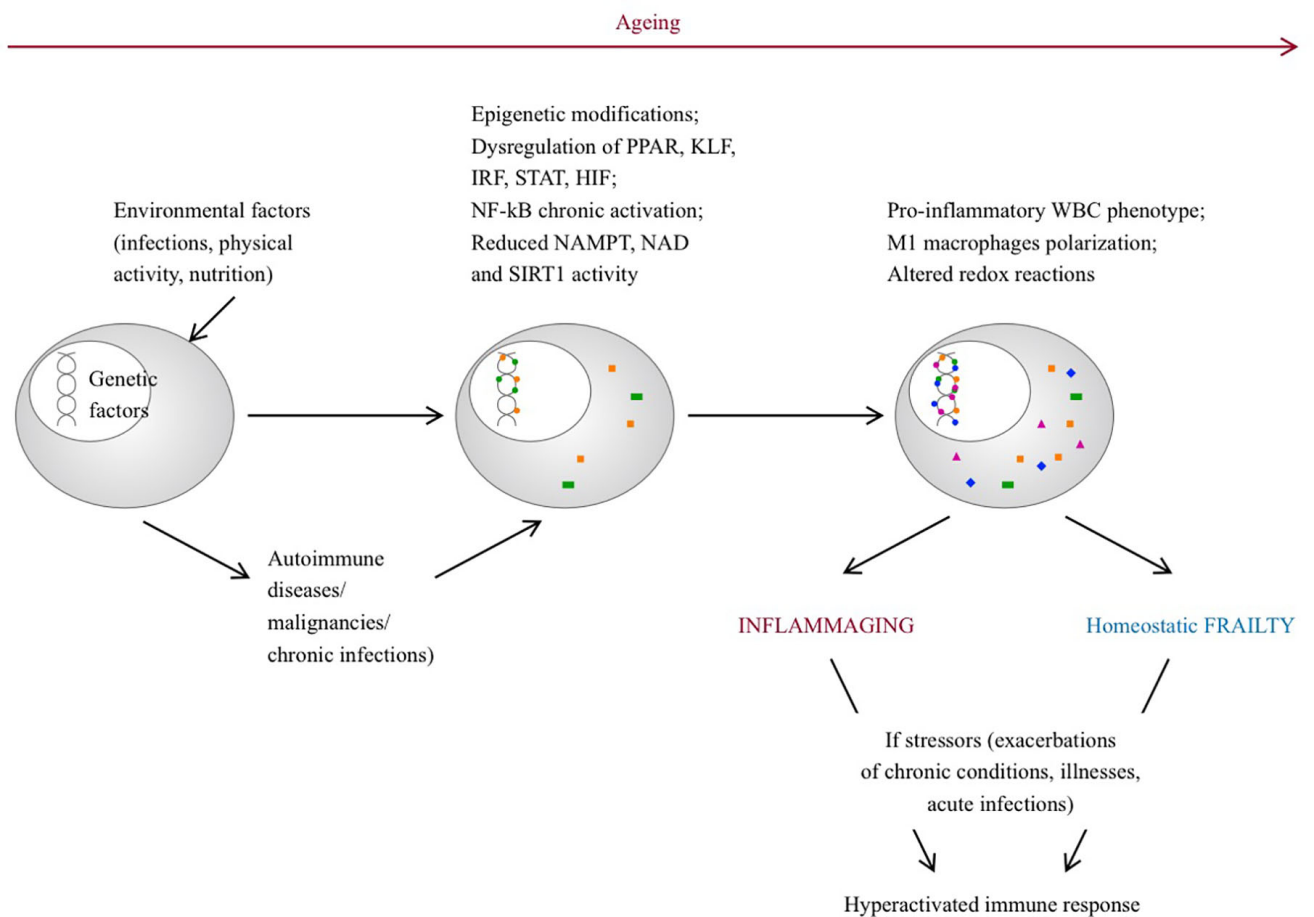
Pathogenetic pathways leading to inflammaging and frailty. With aging, environmental factors can lead to epigenetic modifications, dysregulation of several genes and can promote a pro-inflammatory phenotype of senescent immune cells. On the other hand, rheumatic diseases can develop on a predisposed genetic background, triggered by environmental factors. Chronic inflammation can lead to epigenetic modifications, dysregulation of gene transcription and persistent pro-inflammatory phenotype of immune cells. In both situations, in response to stressor events, the immune system is inappropriately hyperactivated and the ability to promptly restore homeostasis is compromised. PPAR, peroxisome proliferator-activated receptor; FLF, Krüppel-like factor; IRF, interferon-regulatory factor; STAT, signal transduction and activator of transcription; NF-kB, nuclear factor kappa-light-chain-enhancer of activated B cells. HIF, hypoxia-inducible factor families; NAD, nicotinamide adenine dinucleotide; NAMPT, Nicotinamide phosphoribosyltransferase; SIRT1, sirtuin 1; WBC, white blood cells.

The changes associated with frailty largely overlap with aging, where a chronic, sterile, systemic, low grade inflammation, also referred to as “inflammaging,” occurs and leads to increased levels of pro-inflammatory mediators (although within the normal ranges) compared to younger individuals. Genetic, epigenetic and environmental factors contribute to this phenomenon. Senescent immune cells can acquire a proinflammatory phenotype, misplaced nucleic acids can accumulate and trigger an immune response, self-reactive T-cells can be released, infections or gut disbyosis can promote and modulate the inflammatory status. During life, chronic diseases, physical activity, stressors, infections and nutrition may play a role in this process, contributing to the development of different phenotypes. Inflammaging can be considered the consequence of an altered immune function with a dysregulated immune response, similarly to the frail syndrome and although evidence is scarce, it would be intriguing to conceive frailty as an inflammaging-related disease (56) (**Figure 1**). In this regard, the cellular and molecular mechanisms that guide homeostatic frailty are currently poorly characterized. In response to a variety of stresses (trauma, infection, cancer) tissues operate the healing process by chronologically overlapping phases of acute inflammation, resolution of inflammation, proliferation and remodeling, during which macrophages play a crucial role (57, 58). Macrophages operate such functions by exploiting different activation programs, switching their phenotype from an M1- (classically activated macrophages) to an M2- (alternatively activated macrophages) polarized activation. While M1-polarized macrophages are essential to initiate the inflammatory response after injury, by releasing several inflammatory mediators (e.g. IL-6, tumor necrosis factor (TNF)-α, IL-1, and nitric oxide), late switching to an M2 phenotype provides anti-inflammatory cytokines (e.g., transforming growth factor (TGF)-β and IL-10), phagocytosis of apoptotic neutrophils and removal of damaged cells, hence promoting resolution of inflammation and restoration of tissue homeostasis. Perturbation of this dynamic reprogramming of macrophage functions may lead to a failure of resolution of inflammation, eventually resulting in the formation of dysfunctional fibrotic tissue (59). Molecular determinants that affect the macrophage M1 and M2 polarization balance include members of the peroxisome proliferator-activated receptor (PPAR), Krüppel-like factor (KLF), IFN-regulatory factors (IRF), signal transduction and activator of transcription (STAT), nuclear factor kappa-light-chain-enhancer of activated B cells (NF-κB), and hypoxia-inducible factor (HIF) families (60). In particular, whereas NF-κB activation occurs temporarily during a normal immune response, it is chronically activated in the affected tissues of autoimmune diseases, such as rheumatoid arthritis, systemic lupus erythematosus, type 1 diabetes, multiple sclerosis, inflammatory bowel disease (61). Further, accumulation of the p50 NF-κB subunit results in the formation of the transcriptionally inactive p50 NF-κB homodimer, a key regulator of M2-driven inflammatory reactions and inhibitor of M1 macrophage polarization controlling the resolution of the inflammatory response. Accordingly, p50-deficient mice display exacerbated M1-driven inflammation and homeostatic dysregulation (62). In agreement, lack of p50 NF-κB in self-antigen-pulsed unstimulated dendritic cells results in activation of CD8(+) T lymphocytes and induction of autoimmunity (63). More recently the homeostatic role of myeloid-specific p50 NF-κB was strengthen by the observation that its accumulation promotes expansion of myeloid-derived suppressor cells (MDSC) (64), a subtype of myeloid cell primarily beneficial upon restoring homeostasis after inflammation (65). In addition, epigenetic modifications with involvement of miRNAs, histone methylation and acetylation have emerged as regulators of inflammation (66–68). In this regard, reduction of Nicotinamide Adenine Dinucleotide (NAD) levels, as a consequence of the age-dependent decline of the enzyme Nicotinamide phosphoribosyltransferase (NAMPT), represents a major driver of frailty in the elderly (69, 70). This event threatens redox reactions orchestrated by NAD+ (71), as well as the NAD-dependent activity of the deacetylase SIRT1, a major regulator of gene transcription acting through modification of chromatin-associated proteins (72), suggesting that age-related loss of NAMPT/NAD+/SIRT1 activity is likely to undermine the efficiency of antioxidant, metabolic and anti-inflammatory pathways (73, 74).

Interestingly, while the efficiency of the NAMPT/NAD+/SIRT1 system is controlled by a balanced nutritional supply of tryptophan and vitamin B3, which provides the primary and the rescue pathways for the synthesis of NAD, respectively (75), accumulation of p50 NF-κB is promoted by long exposure to bacterial-derived products, including LSP, suggesting that its dysregulation may alter the delicate balance between immune response and immune tolerance in sites exposed to pathogenic microorganism and commensal flora, such as the intestinal tract (76).

Remarkably, the definition of myeloid cell plasticity in pathology has been recently reformulated by the concept of trained immunity, including a set of epigenetic and metabolic events promoting the functional reprogramming of the myeloid cells and myeloid progenitors, in response to secondary stimulation with pathogens, Toll-like receptor agonists and cytokines (77). According to this concept, innate immune cells retain a “memory” of previous microbial and/or traumatic encounters, which influences subsequent immune responses to reproduce similar patterns of activation and inhibition, potentially compromising the ability to promptly activate homeostatic mechanisms.

Therefore, the life events and the environment can lead to the alteration of the efficiency of homeostatic systems, which may likely predispose to a status of “homeostatic frailty” linked to inflammatory, autoimmune and metabolic disorders.

## Tissue Changes and Frailty

**Figure 2:**
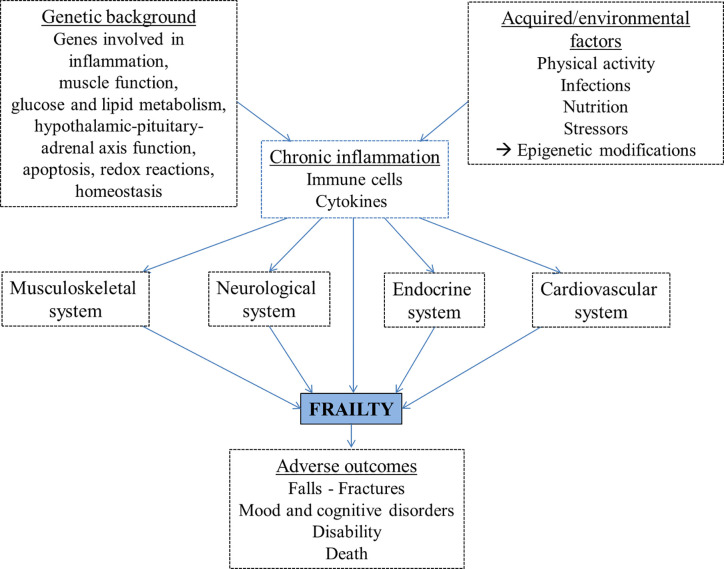
The proposed pathogenetic pathways leading to frailty development include genetic and environmental factors, largely mediated by chronic inflammation, and lead to adverse outcomes.

Inflammation may contribute to frailty directly or indirectly through its detrimental effects on other organ systems (**Figure 2**), for example leading to muscle mass decrease, loss of strength, reduced physical activity, anemia, clinical and cardiovascular diseases and poor nutrition (78–82). The musculoskeletal system plays a key role in frailty, as sarcopenia mostly related to physical inactivity leads to disability, weakness and slowed motor performance. Sarcopenia is the decline in skeletal muscle mass with decreased strength or function. It is caused by age-related alterations of motor neurons and muscle fibers, poor nutrition and physical activity, endocrine changes or chronic inflammation (83, 84).

The endocrine system is also involved in frailty, particularly the hypothalamic–pituitary–adrenal axis (85). Sex hormones, glucocorticoids, growth hormone (GH) and insulin growth factor-1 (IGF-1) are critical for muscle metabolism and trophism. In fact, androgens and, to a lesser extent, estrogens, activate the downstream signaling with transcription of muscle proteins, reduction of protein degradation and myocyte proliferation, either directly or indirectly through increasing the expression of IGF-1 receptors on muscle cells. Glucocorticoid hormone has a catabolic effects on muscle, activating the degradation of ubiquitin-conjugated proteins. GH is the main promoter of body growth in children and exerts anabolic effects in adults, including effects on myocytes, increasing protein synthesis, inhibiting protein degradation and inducing gluconeogenesis. IGF-1, produced in the liver in response to circulating GH, is the main mediator of these effects. Vitamin D is another factor involved in skeletal muscle trophism and lower levels were associated with lower muscle strength, poor muscle function and increased muscle loss (86).

A delicate balance of these hormonal factors determines muscle trophism and strength.

The age-related decrease of estrogen in women and testosterone in older men leads to decline in muscle mass and muscle strength. Circulating levels of the precursor of sex hormones dehydroepiandrosterone sulfate (87) and of IGF-1 (88) are significantly lower in frail older adults. Glucocorticoids induce muscle atrophy by promoting myofibrillar degradation and inhibiting protein synthesis. Higher levels of evening cortisol, 24-hour mean cortisol, and blunted diurnal variation of cortisol have also been associated with the frailty syndrome in the elderly (89). Glucocorticoid therapy is also independently associated with vitamin D deficiency, since steroids interfere with vitamin D metabolism, due to increased renal transcription and expression of vitamin D-24-hydroxylase, which degrades active metabolites such as 25(OH)D and 1,25(OH)D (90). Vitamin D insufficiency is associated with frailty, particularly in older men (91). Vitamin D is a well-known central regulator of bone homeostasis, by determining calcium-phosphorus balance. In addition, vitamin D exerts several extra-skeletal effects, which include muscle tissue trophism, improvement of glucose and fatty acid metabolism and modulation of the immune system, with low levels associated with autoimmune diseases pathogenesis (92). In fact, vitamin D exerts anti-inflammatory properties decreasing monocytes/macrophages recruitment, inhibiting pro-inflammatory cytokines/chemokines expression and enhancing tolerogenicity of dendritic cells (93), thus playing an immunomodulatory role and maintaining immune homeostasis. Not only almost all the immune cells present vitamin D receptor and can be modulated by this hormone, but are also local producers of vitamin D under specific signals (94). Therefore, a deficiency of this hormone may trigger or exacerbate chronic low-grade inflammation, thus enhancing the process that leads to frailty.

The neurological system undergoes structural and functional changes with aging, involving both neurons and microglial cells. Microglial cells can be activated by injury and inflammation, resulting in an hyperresponsive phenotype causing neural damage in response to small stimuli. Frail elderlies have an increased risk of developing dementia or cognitive impairment in the long-term period, with a faster decline associated with increasing frailty. They have also higher risk of developing delirium, and frail patients with delirium have a significant reduced survival compared to non-frail subjects with delirium (5, 95–97). Moreover, frailty is also associated with depression. A meta-analysis showed that each condition is associated with an increased incidence and prevalence of the other, and can be a risk factor for the development of the other (98). Frailty negatively impacts the course of depression (99) and the co-existance of the two conditions is associated with higher mortality (100).

Despite the increasing evidence regarding the multifactorial etiology of frailty, further clinical and mechanistic studies are needed to better understand the complex pathophysiology of this syndrome.

## Frailty in Rheumatic Diseases

Most recently, frailty has moved past being exclusively sought in the geriatric population, and research on this concept has grown in many clinical areas, possibly because of global aging, and recognizing a patient as frail might give a clinical advantage and better guide the management.

In rheumatology, limited data are available and agree on underlining that frailty should constitute a clinimetric assessment and a prognostic factor. This is well represented by both non inflammatory conditions such as osteoarthritis and osteoporosis, and autoimmune diseases such as rheumatoid arthritis, systemic lupus erythematosus, systemic sclerosis, and ANCA-associated vasculitis. In rheumatic diseases, the processes leading to frailty may be related to the disease itself or to the treatment with glucocorticoids and/or with disease-modifying antirheumatic drugs (DMARDs) used in the management of the chronic condition, which may have a dual impact on frailty, on the one hand beneficial on disease activity, on the other hand detrimental, due to side effects, as discussed in each section.

### Osteoarthritis

Osteoarthritis (OA) is a chronic and disabling condition whose prevalence increases with age. Around 20% of middle-aged and older population is symptomatic for OA, while more than 30% presents radiographic signs of OA. As knee OA can lead to disability, a proportion of these patients undergoes joint replacement, which has a mortality risk up to 10% (101). As OA is more prevalent in the elderly, frailty often coexists and the association between the two conditions is significant in both directions. Most relevant to the rheumatologist, frailty predicts mortality in subjects with OA (102). Therefore, it can be considered a new prognostic factor to stratify the population with OA and implement their management.

Several studies have examined the relationship between frailty and OA, all suggesting that the frailty state should be assessed when considering treatment of OA, as it may be essential in targeting therapeutic interventions. **Table 2** illustrates the more recent studies of the prevalence of frailty in patients with OA, while studies on the prevalence of OA in frail subjects remain undetected. Despite measuring frailty with different methods and considering OA at different sites, all these studies found a high prevalence of frailty in OA patients, with crude rates ranging from 24% to 60% (103–108).

**Table 2:** Studies on frailty in osteoarthritis (OA),.

Type of OA	Gender	Measurement of frailty	Results	Ref.
**Hip**	Male	CHS	Prevalence of frail patients: 8%; intermediate: 42%. OA or hip replacement: 1.27 times more likely to be frail.	(103)
**Knee**	Male and female	SOF	Prevalence of frailty: 60%. Severity correlated with prevalence.	(104)
**Hand, hip and/or knee**	Male and female	CHS	Prevalence of frailty 30% (OA any site):. Odds higher if hip or multiple sites affected.	(105)
**Knee**	Male and female	CHS	Knee pain associated with increased risk of frailty.	(106)
**Hand, hip and/or knee**	Male and female	CHS	OA pain associated with higher incidence of frailty (higher risk in women).	(107)
**Hip or knee**	Male and female	GFI	Prevalence of frailty: 24% for knee OA, 33% for hip OA.	(108)

CHS Cardiovascular Health Study Index; SOF, Study of Osteoporotic Fracture index; GFI, Groningen Frailty Indicator.

Modified from O’Brien et al. (109).

Frailty can occur in patients with OA for several reasons. Patients with lower extremity OA, especially hip and knee OA, are likely to reduce their physical activity, which results in loss of muscle mass and increased incidence of falls than age-matched healthy controls (110). OA-related pain also plays a role, as it has been associated with an increased risk of developing frailty compared to people with OA and no pain (103, 107). Pain is directly linked to a loss of physical function and is often associated with cognitive impairment, both of which are observed in frail patients (111). The potential underlying pathways linking OA and frailty are not well understood and studies are scarce. A possible mechanism involves inflammation. Chronic, low grade inflammation occurs in OA and pro-inflammatory markers, as IL-6 and CRP, have been detected in blood of individuals with OA (112, 113). This mild inflammation can induce sarcopenia and decreased physical activity (109).

Intraarticular glucocorticoid injections can be used in hip and knee OA management (114) and are especially useful in reducing pain (115). However, although presenting minimal systemic exposure and toxicity, they can contribute to cartilage loss, and efficacy has been demonstrated only in the short-term. Physical and psychosocial approaches are preferred, at least at early stages, analgesics can be used, while DMARDs are not recommended (114).

Further research is required to understand the pathophysiological pathways and identify potential targets to prevent the development of frailty in OA patients.

On the other hand, frailty might be a risk factor for the development of OA. In frail patients sarcopenia is frequently observed and this can lead to joint instability and increase the likelihood of biomechanical injury. Moreover, frail subjects have a higher risk of falls (18), which could result in fractures, disability and development of post-traumatic OA. Furthermore, higher circulating levels of pro-inflammatory cytokines, such as IL-6, CRP, and TNF-α, are present in frail subjects (43, 49, 116) and a possible accumulation of these mediators in joints has been hypothesized, inducing local low-grade inflammation, cartilage destruction and leading to an altered catabolism of joint structures (109). Older individuals usually have changes in joint cellular composition and on this substrate a pro-inflammatory state might impair the ability of joint repair, with the development of OA. Frailty may therefore be considered as an additional risk factor for the development of OA (109). Of note, in a European multicenter study, Castell and colleagues found a risk of frailty (defined according to Fried’s criteria) from 1.5 to nearly 3-fold higher in patients with OA in any joints, with hip OA associated with a greater risk of frailty compared to knee OA, perhaps due to major disability associated with hip OA and, if multiple joints were involved, the risk was 8-fold higher (105). OA is associated with greater incidence of frailty (104) and with accelerated frailty progression if frailty is already present (103).

### Osteoporosis

Osteoporosis is characterized by reduced bone mass and structural deterioration of bone tissue, resulting in increased bone fragility and higher risk of fractures. The risk of fragility fractures increases with age and in presence of other risk factors, such as treatment with glucocorticoids or diagnosis of rheumatoid arthritis. A history of previous fractures affects the prognosis, with up to 30% one-year mortality for hip fractures (117, 118).

Cook and colleagues reported an association between higher frailty index and lower bone density, assessed by calcaneal quantitative ultrasound in a cohort of more than three thousand men aged 40 to 79 years, after adjusting for age. Possible explanations are related to reduced mechanical loading resulting from sarcopenia, insufficient physical activity, decline in sex hormones, low-grade inflammation, and nutritional deficiencies (119).

In a meta-analysis including almost 100.000 senior men and women from the general population, frailty was associated with a 70% increased risk for a fracture of any type (120). Moreover, the frailty index was higher in women who reported a previous fracture and its changes in 1 and 2 years were significantly greater in women who had suffered a major osteoporotic fracture compared to unfractured subjects (121). As a result, some authors suggest to consider as frail the elderly with osteoporosis-related fractures (122). In addition, as bone density directly correlates with the risk of fracture (123), which is associated with worsening of frailty, we can speculate that dual-energy X-ray absorptiometry (DEXA) may provide information on the potential imminent risk of fracture and, consequently, of frailty. Moreover, as osteoporosis and frailty share etiopathogenic factors such as sarcopenia and inflammatory, hormonal and nutritional alterations, including vitamin D metabolism impairment (5, 124), DEXA could early identify pre-frail or frail subjects. DEXA could also assist the clinician in the decision for the best management for frailty. In fact, when low bone density is detected, an investigation and correction of reversible causes (nutritional, hormonal or inflammatory) is recommended (125) and may also improve frailty risk. Therefore, not only the concept of frailty needs to be implemented in patients with osteoporosis in order improve their management (126), but also considering DEXA in the evaluation of subjects at risk for frailty and treating osteoporosis may be useful in the prevention and management of frailty.

### Rheumatoid Arthritis

Rheumatoid arthritis (RA) is a chronic inflammatory disease which primarily affects the joints with a polyarticular and symmetric arthritis, but it can have extra-articular manifestations such as constitutional symptoms and lung, skin, eye or heart involvement. The clinical management combines the measure of disease activity with composite indexes, a treat-to-target strategy and the use of conventional, biological and targeted synthetic DMARDs (127–129). The use of glucocorticoids, which can predispose to frailty, is recommended only in the short-term, in order to rapidly reduce inflammation, and tapering is recommended as rapidly as clinically feasible, aiming at discontinuation within few months. Otherwise, the dosage should be kept to a minimum, and the reasons for continuing corticosteroid therapy should be periodically checked, in line with patient’s risk-benefit ratio (130). The impact of chronic steroid therapy may be detrimental on bone (131) and muscle (132) and therefore can lead to frailty, but focused research is difficult to perform and is currently lacking. From the few studies available on frailty and RA, described below, disease activity seems the main factor involved in the development of frailty. In this perspective, glucocorticoids may be beneficial when maintained for a better disease control. DMARDs, both synthetic and biological, are essential for steroid sparing and have to be introduced early after diagnosis of RA (130). However, immunosuppression can increase the patient’s risk of infection (133), which can lead to hospitalization and further risk of frailty. Life expectancy of patients with RA is reduced, with a twofold increase in mortality and a decreased lifespan of 7 to 10 years (134). Despite the progresses made in therapy and management of RA over the last decades, mortality rate seems to remain higher compared to general population (135), although an improvement in 5-year mortality has been demonstrated from the introduction of biologic DMARDs into clinical practice (136).

RA may present with features of frailty, such as sarcopenia (137), fatigue (138) and low physical activity, especially when the disease is not well controlled (139). Frailty in patients affected by RA has been evaluated in recent studies. In 2019 a cohort of 210 patients with RA was compared to a healthy control population and, according to SHARE-FI criteria, frailty was more common in the RA population (16.6% vs 8% among controls). Age, comorbidities and disease activity were independently associated with frailty (140). In a cohort of 100 patients younger than 65 years with RA, the prevalence was 15% for frailty and 30% for pre-frailty measured with the SHARE-FI score. Unemployment, higher pain intensity, longer disease duration and higher disease activity were associated with a higher frailty score (141). Frailty appears to be related to disease activity also in an analysis by Tada and colleagues, where the prevalence of frail, pre-frail and normal subjects were 18.9%, 38.9% and 42.2%, respectively. In the subgroup of patients in remission (i.e. with a disease activity score 28 lower than 2.6) 6.7% had frailty, while in those with moderate and high disease activity (disease activity score 28 >3.2) 46.7% were frail (142).

In a cohort of 124 RA cases, baseline frailty defined with the Fried phenotype model predicted significant worsening in physical function assessed with Health Assessment Questionnaire, even when controlling for the effects of disease severity, disease duration, and medication use. The trends were unchanged when the analyses were limited to participants younger than 65 years, suggesting that age is not the only factor involved (143). Salaffi and colleagues recently developed and preliminary validated a cumulative frailty index for RA patients, evaluating nutritional status, weakness, falls, comorbidity, polypharmacy, social activity, pain, fatigue, physical function and depression. Advanced age and high disease activity were significantly associated with frailty, while gender, educational level, disease duration and radiographic damage were not related to frailty in the cohort analyzed (144, 145).

### Systemic Lupus Erythematosus

Systemic lupus erythematosus (SLE) is a chronic autoimmune disease that can affect any organ, with clinical manifestations ranging from mild skin lesions to life-threatening renal, hematological or neurological involvement (146, 147). Management consists of a combined therapy with glucocorticoids and immunosuppressive drugs (148). Severe manifestations usually require high-dose glucocorticoids, with a potentially greater impact on the development of frailty. Moreover, as SLE affects fertile women, glucocorticoids are introduced early in life, with greater risk of long-term complications. Immunosuppressive drugs usually allow steroid tapering, but some of them are burdened with severe side effects, as cyclophosphamide which can induce ovarian failure (148), increasing the risk of frailty development. The survival rate in SLE has been increasing over time, but the mortality risk remained stable at over 3 times higher than that of the general population (149). In 2016 Katz and colleagues (150) assessed frailty in a population of 152 women with SLE (mean age 48 years) using the Fried’s phenotype criteria and 20% of the sample was classified as frail, 50% as pre-frail. Exhaustion, weakness and inactivity were the variables most commonly present (45%, 31% and 29%, respectively). There were no differences in age, race, education, duration of the disease, or smoking history by frailty classification. Steroid use was more common among pre-frail and frail women. There was a significant worsening in ratings of disease activity, damage and pain as frailty state moved from robust, to pre-frail, to frail. Frail women had significantly worse physical functioning and were more likely to have cognitive impairment compared to both robust and pre-frail women. Although mortality was a rare outcome, frailty was linked to a higher risk of death, estimated around six times higher than in robust women.

In 2019 Legge and colleagues described a frailty index for SLE, using data from the Systemic Lupus International Collaborating Clinics (SLICC) inception cohort (151) in 1682 SLE cases (89% females, mean age 35.7 years, mean disease duration 18.8 months) (152) and reported that higher baseline SLICC frailty index values were associated with increased mortality risk, even after adjusting for age, sex, steroid use, ethnicity and baseline disease activity. Therefore the SLICC frailty index might help to explain the heterogeneous health outcomes in patients with SLE and can be a useful tool to provide prognostic information and to predict future mortality risk.

### Systemic Sclerosis

Systemic sclerosis (SSc) is an immune-mediated disease characterized by cutaneous thickening, progressive fibrosis of skin and internal organs and vasculopathy, leading to the highest mortality in rheumatic diseases, estimated to be four-fold higher than general population (153, 154). Management aims at reducing or stabilizing disease manifestations. Vasodilators are used for vascular-related complications, immunosuppressive therapy for inflammatory manifestations and interstitial lung disease, while glucocorticoids have to be avoided or used at low dosage, due to the risk of scleroderma renal crisis (155, 156). Therefore, in SSc patients, if vasodilators only are used, therapy does not have a great impact on frailty, while if immunosuppressive drugs need to be introduced, side effects (157) can worsen the condition of patients affected by a debilitating disease.

A cumulative frailty index has also been developed in systemic sclerosis, according to the procedure described by Searle and colleagues (34). In this study, 44 items were selected from the Canadian Scleroderma Research Group (CSRG) Registry as health deficits. The frailty index was applied to a population of 1372 CSRG patients. The score was higher in diffuse rather than limited disease, correlated with the Rodnan Skin Score and with the damage assessed by physicians. It increased with age, but not linearly, reflecting the fact that patients with SSc may be vulnerable even at younger age and that frailty, damage assessed by physicians and age were predictive of mortality. Irrespective of age, disease type, or time since diagnosis, the risk of death was higher with higher frailty index scores (158).

Frailty in SSc has also been studied in relation to interstitial lung disease (ILD) (159). Measured using a 42-item index, frailty was found to have a prevalence of 55% in patients with SSc-ILD and to be strongly associated with dyspnea. The frailty index did not significantly differ from that in a control population with ILD not associated to a connective tissue disease, even though SSc patients had a significantly younger age, indicating that chronological age significantly underestimates biological age in SSc patients and that the concept of frailty could allow a more accurate prognostic evaluation than demographic and disease-specific parameters alone.

### ANCA-Associated Vasculitis

ANCA-associated vasculitis (AAV) includes different forms of necrotizing vasculitis affecting predominantly the small vessels, which have in common the serum positivity for antineutrophil cytoplasmic antibodies (ANCA), the frequent lung involvement and a pauci-immune crescentic glomerulonephritis, which potentially leads to end-stage renal disease. The prevalence of AAV is estimated around 100 per million people. The 5-year survival rates range from 45 to 97% (160).

Severe cases involving lung or kidney are treated with high-dose glucocorticoids and immunosuppressive drugs, either cyclophosphamide or rituximab, for remission induction. The maintenance regimens consist of low-dose glucocorticoids and chronic immunosuppressive drugs (161). On the one hand this management reduces chronic inflammation and allows good outcomes and survival (162), on the other hand it can lead to sarcopenia, bone loss, higher risk of infection (163) and frailty development in the long-term.

McGovern and colleagues described a population of 83 patients aged 65 or over with a diagnosis of AAV, studying the risk factors for mortality (164). They used the Canadian Study on Health and Ageing Clinical Frailty Scale (22) to measure frailty. Age, very high CRP values and baseline frailty score were independently associated with mortality. For each additional point on the frailty score, the risk of death approximately doubled, even when adjusting for age, sex, ANCA status, renal function and CRP values. Patients with a lower vs higher baseline frailty score (≤3 vs ≥4) had no differences in time to remission or time to relapse, but in the frailer group a greater proportion of patients had adverse events, longer in-hospital stay and mortality was significantly higher, with a five-year survival of 47% vs 90% respectively.

## Natural History and Management of Frailty

Although the prevalence of frailty gradually increases with age (11), it has been demonstrated that older people may have dynamic transition of their frailty status over time, with 25% and 3% among pre-frail and frail patients improving to a robust state (165). Therefore, frailty is not a static state, some deficits can be reversed and an early intervention is preferable. In longitudinal population-based studies a range of 8.3%–17.9% of older adults spontaneously improved their frailty state in the follow up years (166–168). Poor prognostic factors for improvement were older age, poor handgrip strength, weight loss, poor physical activity, hospitalizations, previous cancer or stroke, lung disease, lower cognitive function, diabetes, osteoarthritis, low albumin levels and high IL-6 levels. Female sex, being married and having a higher socioeconomic status were protective.

Currently, there is no standard of care for frailty. Evidence supporting interventions and strategies to reverse or minimize frailty varies across the studies; however a high level of evidence is currently lacking. Interventions for the frailty syndrome should aim to prevent, delay, reverse or reduce the severity of frailty and prevent or minimize adverse health outcomes in those whose frailty is not reversible.

Among the prognostic factors involved in frailty, muscle strength is one which could be improved with training. In fact, exercise is the intervention that has shown to result in some benefit in frailty (20, 169). Exercise has physiologic impacts on many systems, particularly musculoskeletal, endocrine and immune systems (9). However, the evidence of achieving good outcome with exercise is low or very low. Moreover, the optimal program for physical activity is not known, with studies showing differences in frequency, intensity, type and duration of exercise (170).

Another aspect of frailty susceptible to intervention is nutrition. The correction of nutritional deficiencies, including micronutrients, improves physical performance but not body weight; in a meta-analysis the evidence was reported as of moderate-level certainty, but the study had several biases (171).

A pharmacological approach has been evaluated in a few studies. As a decline in sex hormones occurs with aging (172) and low levels are associated with frailty (87), while supplementation improves performance of organs involved in frailty as the muscle and the bone (173–175), hormone replacement therapy with testosterone (in men) or estrogens (in postmenopausal women) has been evaluated. It obtained some effect on frailty (176), particularly on muscle strength, but treatment was burdened with significant systemic side effects, mainly cardiovascular for testosterone (177), oncogenic for estrogen supplementation (178).

Vitamin D has a favorable pharmacological and safety profile, but its clinical utility in frailty has to be better investigated (9). The use of statins, known to have anti-inflammatory effects (179), had no association with reduction of incidence of frailty (180).

An integrated intervention was implemented in Japan, where people were screened for frailty in primary care. Subjects identified as pre-frail or frail were referred to a group community program involving physical activity, nutrition, and social participation, with substantial functional improvements in the population in a 10-year period (181).

## Discussion

Rheumatic diseases affect patients at any ages and often lead to progressive reduction in physical capacity, increased vulnerability to adverse events and thereby to frailty. Furthermore, current medical treatments allow a longer survival, thus possibly increasing the incidence and prevalence of frailty in these patient populations. Our overview on frailty in rheumatic diseases underlines how important it is considering this parameter in patient evaluation. In fact, age was non-linearly correlated to frail state, and the association between frail status and unfavorable outcomes persisted after adjustment for age (140, 141, 143, 150, 158, 164). Moreover, some analyses have been performed in young populations, as it was in a RA cohort (141) and in SLE patients (150) and frailty was associated with high disease activity, implying a key role of inflammation. Accordingly, immunosuppressive therapy could play a major role in preventing frailty. On the other hand, in AAV it was shown that patients with a lower vs higher baseline frailty score had no differences in time to remission or time to relapse, but in the frailer group a greater proportion of patients had adverse events, longer in-hospital stay, and mortality was significantly higher (164). Therefore, while a more aggressive therapy could be necessary early in the course of rheumatic diseases also to prevent frailty, in an already frail patient it could jeopardize any expected advantages and a more conservative management might be preferable. Both rheumatic diseases and frailty can influence each other and their severity may vary over time. Therefore, as the disease activity in rheumatic patients is evaluated regularly and therapy modified accordingly, frailty should also be assessed periodically. The introduction in rheumatology of a frailty score would be a valuable tool to better understand the patient overall health status and to perform a proper prognostic assessment. The challenge is to develop a standardized frailty definition and screening tool, in order to allow an homogeneous assessment and risk stratification for each disease. A disease-specific scale of frailty should be defined for each rheumatic condition and would likely be a proper way to address the issue. In fact, it would include different or additional factors compared to Fried’s criteria, that would better predict predisposition to poor outcomes. For example, a systemic sclerosis-specific scale might include cardio-pulmonary evaluation, while in rheumatoid arthritis joint-related problems should be considered. Moreover, the assessment of disease-related complications is routinely performed during clinical evaluation, while additional tests are needed for Fried’s criteria, and this can limit the feasibility in clinical settings. Pilot studies are reported in this review, but further analyses on larger casuistries are needed to validate the FI proposed or to identify other scales. The introduction of frailty scales for rheumatic diseases may allow a more complete evaluation of the patient in the clinical setting. In addition, frailty should be evaluated in clinical trials. Evidence for treatment and current management recommendations are based on clinical trials, but frail subjects may not comply with the numerous study requirements or, if enrolled, they may have an increased dropout rate due to adverse events. Considering frailty in clinical trials is particularly challenging, due to the heterogeneity of variables involved, even within the same rheumatic disease, but an effort must be made in this direction. In observational and, most importantly, in prospective and interventional trials on drugs, frailty have to be defined and investigated. Moreover, in the analysis of outcomes, the importance of diagnosing frailty and the impact of potential interventions to decrease risks have to be evaluated. Patients stratification according to frailty state could allow a better management, considering not only the specific disease, but taking into account also the patient’s global health. The risk-benefit ratio of glucocorticoids and different immunosuppressive drugs should be investigated in-depth in frail patients affected by rheumatic diseases, as they can be more vulnerable to adverse effects, and a deeper insight of the consequences of treatment on frailty could help clinical decisions. Furthermore, it would allow to establish personalized programs to prevent frailty, or reverse or reduce its severity and to manage the associated complications, with an enhancement of physical, psycho-social and nutritional support. Finally, assessment of frailty should be included in real-life studies, for a better estimate of the impact of proposed medical treatments.

In conclusion, frailty is an emerging concept in rheumatology and should be implemented with the definition of disease-specific scales. In addition, clinical trials should be performed to study the impact of frailty on different rheumatic diseases, and, on the other hand, of rheumatic diseases and immunosuppressive drugs on frailty and related adverse events. As more treatment options are now available for rheumatic diseases, the need for tailored therapy has become increasingly relevant. Further research on frailty could allow to identify the best treatment approach for each patient, aiming at minimizing drug-related adverse effects while optimizing effectiveness and patient outcomes.

## Author Contributions

Study concept and design: CS, AS. Drafting of the manuscript: FM. Critical revision of the manuscript for important intellectual content: FM, AS, CS. All authors contributed to the article and approved the submitted version.

## Conflict of Interest

The authors declare that the research was conducted in the absence of any commercial or financial relationships that could be construed as a potential conflict of interest.
